# Medical Society of the County of Erie

**Published:** 1910-04

**Authors:** Franklin C. Gram


### SOCIETY PROCEEDINGS.

### Medical Society of the County of Erie

Reported by FRANKLIN C. GRAM, M. D., Secretary.

THE regular meeting of the Medical Society of the County of Erie was called to order by the president, Grover W. Wende. February 21, 1910, at 8.30 p. m., in the rooms of the society of natural sciences Buffalo Library Building. On motion, the reading of the minutes of the last regular meeting was dispensed with, inasmuch as they had been published in full in the Buffalo Medical Journal.

The minutes of the council meetings held January 7 and 10, and February 7, 12 and 21, 1910, were read and, on motion, approved.

Dr. T. H. McKee, Chairman of the Committee on Membership presented a list of applicants for membership, each of whom had been previously reported upon favorably by the council.

On motion, the secretary was directed to cast the ballot of the society for each candidate as the name was read by the chairman.

This being done, the following were declared duly elected to membership:

George H. Westinghouse, 541 E. Eagle St.; M. A. Richter, 428 Goodyear Ave.; Walter S. Goodale, 90 N. Pearl St.; George R. Critchlow, 505 Norwood Ave.; William A. MacPherson, 188 Morgan St., Tonawanda, N. Y.; Edward H. Storck, 225 E. Utica St.; Christian A. Weinbach, 2123 Bailey Ave.; A. T. Bull, 615 W. Ferry St.; Edward N. Pfohl, 453 Niagara St.; Archibald D. Carpenter, 346 Pennsylvania St.; Burt J. Maycock, 560 Delaware Ave.; Charles J. Mengis, 88 Goodell St.; Joseph Spangenthal, 115 Bidwell Parkway; Carroll J. Roberts, 393 Porter Ave.; William Linton Phillips, 449 Franklin St.; George J. Hearne, 197 Auburn Ave.; Harvey R. Gaylord, 113 High St.; William Gaertner, 194 E. Utica St.; Frank A. Valanti, 192 Georgia St.; Edward Murray Tracy, 567 Ridge Road, Lackawanna; Walter J. M. Wurtz, 30 Kenmore Ave., Kenmore; Emil Lustig, 644 William St.; James J. McFadden, 173 Auburn Ave.; Edward D. Gibson, 659 Ellicott St.; John Chalmers, 328 W. Ferry St.; William H. Marcy, 32 W. Utica St.; Simon Eschelman, 421 Franklin St.; Dennis J. Constantine, 528 West Ave.; W. G. Grove, 723 Prospect Ave.; Mary Innis Denton, 230 Potomac Ave.; George B. Stocker, 901 Genesee St.; Arthur B. Allen, 868 Main St.; Clarence A. Tyler, Alden; Clinton A. Sage, 213 W. Ferry St.; Charles E. Long, 68 Niagara St.; Albert Frey, 398 Jefferson St.; Albert Brennan, 62 North Ogden St.; Harry H. Ebberts, 890 Humboldt Parkway; Floyd S. Crego, 478 Delaware Ave.; Theodore E. Flemming, Union Road, Gardenville; James W. Charters, 540 Walden Ave.; J. Henry Dowd, 40 N. Pearl St.

At the completion of the election, the president called attention to the fact that these made forty-two (42) members elected at the first meeting of this year. The announcement was received with applause.

Dr. Wall moved that Dr. A. T. Bull be elected to honorary membership. The motion was adopted, but, under the rules, it must lie on the table until the next meeting, when it may be taken up for adoption.

Dr. J. H. Grant, chairman of the Board of Censors, made his report as follows:

*To the Medical Society of the County of Erie:*

Gentlemen—Since the last report, December 20, 1909, the Board of Censors have investigated and acted upon the following cases: The Board met on January 12, 1910, and formally organised by electing John H. Grant as chairman and F. E. Fronczak as secretary for the year.

The case of Elmer G. Hickox, upstairs, 491 Washington Street, Buffalo, who distributed cards reading "Strictly confidential explanations"—Elmer G. Hickox—Buffalo post office," whose business was believed to be that of an abortionist, was referred to the District Attorney in December, 1909, and Mr. Dudley informed the chairman that he would take the matter up with Superintendent Regan for investigation, and that he would advise with the postal authorities. Nothing further has been heard from the case.

In December, a letter enclosing an advertisement of Doctor John H. Dye of Buffalo, was received from Dr. Augustus G. Downing, First Assistant Commissioner of Education, Albany, and referred to Mr. John Lord O'Brian, U. S. District Attorney, Buffalo, who wrote saying that he had referred the matter to the postoffice inspector of this district for immediate investigation, and if it be a violation of the statute, a speedy prosecution will be instituted. As Dr. Dye had been dead for five years or more, evidently a Medicine Company in Buffalo was fraudulently imposing upon the public. The advertisement was cut from an Ohio newspaper.

On January 14, 1910, a warrant was sworn out in the city court, against D. L. Black or Fekete, of 1922 and 1931 Niagara Street, for practising medicine illegally. The warrant was sent to Station 13, about 4 p. m., but no effort seems to have been made to serve it immediately, and about 11.30 p. m., your chairman was notified that Black had suddenly absconded to Bridgeburg, Canada.

On January 13, 1910, Anna Wozesinska, of 144 Person Street, Buffalo, was arrested, through the efforts of a member of the Board, Dr. Fronczak, for attempting to diagnosticate and to treat an incurable disease of a child with a lot of drugs or what not (the drugs on exhibit before the court looked like a lot of horse medicines in beer bottles, etc.) and on January 21, the woman was convicted by Judge Nash and fined $50.00, which sum was paid, and your chairman has applied to the Board of Councilmen for a warrant on the City Treasurer for this amount, under Section 174 of the Public Health Law.

On February 9, 1910, Mrs. (Dr.) Matie E. Lane of 122 Whitney Place, Buffalo, was placed on trial in the criminal part of

Supreme Court before Mr. Justice White, under an indictment for the unlawful practice of medicine. The defendant claimed to be a spiritualist and that she was exempt from prosecution, because of the religious tenents of her church, and this argument was used strenuously during the trial, the defense placing on the stand certain noted professors of the cult from Rochester and Hamburg. Medicine was given and proven in this case, but the defendant claimed she prescribed it under the religious tenets of her church while in a so-called trance, she being the medium of one Dr. Springsteen, dead some dozen years, and, in his lifetime, the dispenser of certain female quack medicines, at Cleveland, O.

The result was a disagreement of the jury—eight of them voting for conviction, and four for acquittal. This is a vital case for the interest of the profession in this state, and it behooves this society to use all its influence on the District Attorney of this County that this case be promptly retried and vigorously handled by his office.

On February 11, 1910, Albert Dellenbaugh, late of Elm Street, Buffalo, pleaded guilty to the charge of unlawfully practising medicine, not having been registered, and was fined $250.00 in criminal term of Supreme Court before Justice White. This man was prepared to pay a much larger fine, but the justice thought $250.00 as a fit punishment to the crime. Dellenbaugh promised to leave the state and go to California. The chairman has applied to the County Board of Supervisors for a warrant on the County Treasurer for the amount, under the law.

On February 16, 1910, a warrant was sworn out against the "Berlin Medical Offices," located at 207 Main Street, Buffalo, for practising medicine, and advertising in a Buffalo newspaper in violation of law. One, Emil Salzer, the manager, was arrested and arrainged in City Court, February 17. Adjourned to February 18 and again adjourned to February 23, 1910. The chairman had the doctor of the concern, one John Treskow, who is registered by the Regents as a graduate of the New York University, 1882, subpenaed in the Sulzer matter and after giving his testimony, a warrant was sworn out for his arrest and he was arraigned in City Court, February 18, for practising medicine under an assumed name—that of the "Berlin Medical Offices." His case was also put over to February 23, and the chairman asked that the bail in his case be made substantial. We hope to win these cases, and if we do, it should end the career of medical quack companies in Buffalo. Judge Brennan has both cases, and it is up to members of the society to lend their moral support in strengthening him to interpret the medical law as it was intended to be interpreted.

There are several cases pending investigation and future action, if warranted.

Respectfully, for the Board of Censors,

(Signed) JOHN H. GRANT, Chairman.

The foregoing report was adopted and the thanks of the society tendered to Dr. Grant and the Board of Censors.

Dr. Grant offered a resolution which was adopted as follows:

WHEREAS, A certain spiritualist, claiming the right to give drugs, as the medium of a certain quack doctor, deceased, through the "religious tenets of her church," as a defence in her trial before a recent term of the Supreme Court of Erie County indicted for illegally practising medicine, and

WHEREAS, This defence resulted in a disagreement of the jury, and vitally affects the enforcement before the courts of the new medical law known as Article 8, Chapter 49, Sections 161 to 174 of the Public Health Law, *Therefore be it*

*Resolved* by the Medical Society of the County of Erie, in session at Buffalo this 21st day of February, 1910, that a copy of this resolution be forwarded to the Secretary of the State Medical Society with a view of its subject matter being considered by the Committee on Legislation, to the end that the law be amended prohibiting any person from practising "the religious tenets of his or her church, from giving or prescribing drugs or the use of surgical instruments."

Dr. F. Park Lewis, chairman of the Committee on Legislation, made a verbal report of the committee's work, especially calling attention to the anti-vivisection bill now before the state legislature. He said that a resolution in opposition to this bill had been drawn and would be sent to the members of the Senate and Assembly from Erie County and also to the Chairman of the Judiciary Committee which had this bill in charge.

The report of the committee was adopted.

Dr. Lytle moved that the secretary be instructed to publish this resolution of condemnation in the daily papers. Dr. Stockton opposed this as useless. Dr. Thornton moved an amendment to send the bill and resolutions to those members desiring the information. Both resolution and amendments were then withdrawn.

Dr. Bonnar, Chairman of the Committee on Contagious Diseases Hospital, made a detailed report of the work done by his committee since the last meeting. The report, including communications, is as follows:

*The Honorable Board of Councilmen:*

MR. HENRY ADSIT BULL, Chairman of Hospital Committee.

Gentlemen—In reply to your communication of the 5 inst., relative to the adaptability of the area of either or both of the proposed sites for the hospital for contagious diseases, I will briefly relate my own conclusions from available data and conference with my confreres on the various questions submitted.

The rectangular tract of land, some five acres, while larger in area, was less adapted for the hospital uses than the irregular tract. In fact the former did not appeal to any of our committee, nor would it afford equal facilities with the lot of smaller area, comprising, with all possible additions, some four acres.

Five acres is the minimum acreage such hospitals require for satisfactory treatment of the various contagious diseases to be segregated within one enclosure. It would, therefore, make it difficult to comprise within the four acres the entire hospital plant. The approach from three sides with the additional area of the streets, ground and necessary space for expansion, either for the buildings or grounds for such needed recreation.

The outer "sanitary zone" would, therefore, rather be the true circle of isolation, that gave actual immunity to the outside public. This outer zone would afford grounds for landscape work and beautifications with shrubbery, flowers and lawns as appeal to the esthetic sense and give that finish which attracts the public,—a paramount feature in securing the fullest efficiency through such institutions.

While five acres is regarded as the minimum area required for hospitals of this nature, ten or more is preferred if such can be secured, centrally located. Statistics show that the minimum zone is inversely as the distance from the hospital—the closer, outside of the sanitary zone, showing fewer cases of contagion.

The Fulham smallpox hospital furnished, however, very striking evidence of a contrary nature. As a result of this, smallpox hospitals were considered dangerous within a quarter of a mile of sparsely settled, or one half a mile of denser population, which conclusion would effectually eliminate our present quarantine hospital grounds from the field of available sites for the proposed hospital for contagious diseases.

Present provision for 250 beds, with possible enlargement of one pavilion and erection of another, to accommodate an additional might be possible on the four acre tract. This would obviously use up the land to such an extent as would seriously diminish the outside accommodations, and thus detract from the practicability of this proposed site of some four acres.

Forty feet being the minimum distance from lot lines and streets for the hospital structures proper, and for the buildings from each other, yet sanitary rules prescribe from 40 to 100 feet, in each instance. Comprising with the greater distance, we consider 60 feet between the buildings a conservative minimum, while 40, from lot lines, might be suitable. Modern rules prescribe 144 square feet for floor area, and 2,000 cubic feet of air as requisite for each patient. It is obvious, therefore, that liberal dimensions are a prime factor in hospitals of this nature.

To facilitate your analysis of this proposition, I am pinning upon the chosen, or preferred site, pieces of paper figured to the same scale as the map, the outside dimensions of the various buildings needed in such a plant. I also enclose a tracing of a hospital for such purposes in the city of Boston. The larger buildings will be respectively for scarlet fever and measles, each giving space for 100 beds, while the smaller pavilion, designed for diphtheria, would accommodate 50. The construction of the latter could be two stories of half the length of the others, but having room for similar size of extension, if required later.

Similarly, either of the other diseases might demand, at some later date, additional buildings.

The conclusions from the foregoing premises suggest the wisdom of providing a larger tract of land than is comprised in the smaller, yet preferable, selection submitted. While fully aware of the great advantage of central location, yet mindful of the great future of our city and that we must build for the future, it appeals to me that amplitude being a first requisite and the future of our city being an object of vital interest in the selection of such a site, the interests of Greater Buffalo will best be reached in this matter by procuring greater acreage, with an unrestricted outlook and air space, with such room for expansion as the prospective growth of our city suggests.

The arrangement of the paper outlines, being simply an adjustment to the area of the acreage and lot lines, I suggest any other arrangement that might appeal to one trained in landscape and architectural fitness. In conclusion, I would express our appreciation of your thoroughness and desire to meet this issue in its fullest relationship to the pressing demands, now imminent in the City of Buffalo.

Respectfully submitted,
John D. Bonnar,
Chairman of Committee.

Membership of committee on hospital for contagious diseases for the Medical Society of the County of Erie:

Drs. Henry R. Hopkins, Charles A. Wall, P. W. VanPeyma, Herman E. Hayd, L. G. Hanley, DeLancey Rochester, A. H. Briggs, W. C. Callanan, Francis E. Fronczak (Deputy Health Commissioner), Franklin C. Gram, Secretary; W. D. Greene, H. E. Matzinger, Edward Clark, John D. Bonnar and Ernest Wende, (Health Commissioner).

February 10, 1910.

Mr. Henry Adsit Bull,
Chairman of Hospital Committee of Council.

Dear Sir—As desired by you, I have prepared some additional data, pertaining to the "zone" features, of influence around such hospitals for contagious diseases. Trusting that the results may prove the wisdom of all proper familiarity with this timely subject and that the fruition may not be far removed from the present, I am

Yours very truly.
John D. Bonnar.

SUPPLEMENTARY DISCUSSION ON HOSPITAL.

The efficient methods, under present hospital equipment make these institutions attractive to the parent and public. In Brighton, Eng., 80 per cent. of contagious diseases are given hospital care.

Similar preference will speedily follow in other cities, where modern hospital facilities, coupled with attractiveness of surroundings, are provided—prerequisites in convincing the parent to yield, even temporarily, the custody of his child.

Statistics show that modern hospital care of contagious diseases, reduces the mortality to 1-5 for like numbers under domestic treatment, even where the hospital cases are relatively poorer nourished, in many instances, than are home patients.

The "sanitary zone" of 40 feet, previously mentioned, as giving immunity to neighboring people, should be rather reckoned from the *limits* of ground used for the hospitals proper, or for recreation purposes. Convalescents, while able to enjoy outside liberty, within the enclosure, are still liable to infect others, hence are still quarantined. It therefore becomes apparent that the "sanitary zone" should be measured from the outside limits of the inner enclosure, which includes all necessary play—while increasing the air space and outlook, yet afford no opportunity for any proper expansion. However, with the desire to use available sizes, we have placed the various buildings, considered necessary in such equipment, within the smaller tract. While it might be utilised for present demands, it would afford such restricted lawn and grounds, for exercise and sports, essential for advanced convalescents in quarantine, its small area greatly restricts such useful functions.

While the fuller protection of the community requires such restraint, needless to say, the children, under enforced isolation, require attractive exercise to keep their minds and bodies from deterioration.

In round numbers, such hospitals call for one bed for every one thousand inhabitants of the city. Realizing the fact that many homes afford ample room for care and isolation of sick members of their families, it will so materially lessen the total patronage of the hospital, provision for three hundred, or better even three hundred and fifty, would give our city, at present, comfortable quarters for prospective patients.

JOHN D. BONNAR.

The President called upon Dr. Stockton to report upon the invitation to be extended, through the State Society, to the American Medical Association, to meet in Buffalo in 1911. His report was favorable.

Dr. Cora B. Lattin presented an outline of the plan of the American Medical Association women's work for the prosecution of public health work. This work has been actively taken up in places, with gratifying results, and has started in Buffalo. A hygiene committee has been appointed and is actively at work. An initial lecture has been given before a large audience of women, by Dr. Stockton, who asked for the appointment of a committee to act with a committee of the Woman's Education Work, for the purpose of furthering a plan, and also to hold public meetings for public health education.

Dr. Wall moved that the request of Dr. Lattin be referred to the committee on publicity which is one of the committees the president is authorised to appoint. The motion was carried.

Dr. McKee moved that the entertainment committee be authorised to draw upon the treasurer for the amount of expense incurred in providing a collation and holding this meeting. The motion was adopted.

The scientific part of the program was then reached.

Dr. Lyon, who was on the program for a demonstration of lantern slides, gave way to Dr. Matthew D. Mann, who was to read the paper of the evening, and said that he would gladly give up his entire time to the discussion of Dr. Mann's paper, as it might otherwise come too late for a proper consideration of the important subject.

Dr. Mann then read a very interesting paper on the "Division of Fees." (This paper is published elsewhere in this issue).

At the conclusion of the reading of Dr. Mann's paper a discussion followed. Dr. Hopkins thought that if the standard of medical education was revised, the number of grafters referred to in Dr. Mann's paper would be diminished. Dr. Schroeter said that this division of fees also extended to the practice of midwifery. Dr. Bonnar suggested that, instead of removing by force, it should be done by natural means. Instead of having only one consultant, have more, which would prevent collusion. There should also be more equity. Dr. Benedict said that during a practice of twenty-two years he had never been approached for a division of fees.

Dr. Wall said that the American Medical Association had already taken action by the adoption of a code of ethics by which a division of fees is prohibited, and thought further action by this society was not required.

Dr. Woodruff said there were two sides to this question. The general practitioner is not the only sinner; but the corporation surgeon who undertakes to treat the injured for a mere pittance, and the physician who undertakes society work should be eliminated. Dr. Pettit thought that the publication of Dr. Mann's paper itself would be a proper remedy. Dr. Blaauw called attention to the legalising of optometry; and Dr. Lewis agreed with him that the student should be taught to recognise the disease for the purpose of sending patients to specialists instead of teaching them to treat such patients. Dr. Lyon thought publication would be the cure; names of specialists should be published.

Dr. Allen A. Jones said that a committee to be appointed to consider this matter, should also consider Dr. Mann's paper and Dr. Stockton's presidential address before the society. Dr. Cohen said a chair of economics should be added to those already existing in medical colleges.

Dr. Pryor moved that the president appoint a committee to investigate this entire subject and particularly its causes; also that Dr. Mann's paper be printed in the BUFFALO MEDICAL JOURNAL, and a reprint sent to every practitioner in Erie County.

Dr. Cary supported Dr. Pryor's motion, stating that the committee should investigate the entire practice and formulate a plan for action. The motion was then adopted.

Dr. Stockton also favored the appointment of a committee of investigation; and in order that Dr. Mann may realize the appreciation of his paper, that it be given the endorsement of the society; and, that the society free itself of the stain, he moved that the society show its appreciation by a rising vote, which was done.

Adjournment then followed, after which a collation was served.

